# Comparing the impact of the Sport Education model on student motivation in Kuwaiti and American students

**DOI:** 10.3389/fpsyg.2024.1334066

**Published:** 2024-02-16

**Authors:** Omar Albaloul, Pamela Hodges Kulinna, Hans van der Mars

**Affiliations:** ^1^Mary Lou Fulton Teachers College, Arizona State University, Mesa, AZ, United States; ^2^College of Education, Kuwait University, Kuwait City, Kuwait

**Keywords:** Sport Education, traditional, physical education, motivation, intervention

## Abstract

**Background:**

Sport Education (SE) stands as the most researched pedagogical model in physical education. While researchers have consistently underscored its capacity to improve student motivation in physical education, a gap remains concerning its implications in Arab nations. Moreover, no studies have been identified comparing the outcomes of SE between the U.S., where the model originated, and other countries.

**Purpose:**

This study was conducted to (1) determine the impact of SE on Kuwaiti students' motivation, (2) determine any differential effects of SE on Kuwaiti and American students' motivation, and (3) explore students' perceptions of SE in both countries.

**Methods:**

A replicated mixed methods quasi-experimental pre-test and post-test design was used. Participants were 33 secondary school students (nine girls and 24 boys; ages 11–12) from two classes in southwestern US, and 37 secondary school students (12 girls and 25 boys; ages 10–11) from two classes in Kuwait. Both sets of classes across the two countries were instructed by the same teacher who was trained in teaching SE. Student interest/enjoyment, perceived competence, effort/importance, and pressure/tension were measured using the Intrinsic Motivation Inventory instrument (IMI). Student perceptions of SE were assessed using a group Semi-structured interview. Quantitative data were analyzed using repeated measures and mixed ANOVAs followed by *t*-tests. Qualitative data were analyzed using a thematic analysis technique.

**Results:**

Kuwaiti students' perceived interest/enjoyment, perceived competence, effort/importance, and pressure/tension scores significantly improved in the group that taught using SE only. Comparing the impact of the SE on students' motivation between the two countries showed no significant differences. The interview data reflected further support for the IMI results.

**Conclusion:**

SE can be effective in increasing Kuwaiti students' motivation in physical education. This motivating effect of SE was observed consistently across both Kuwaiti and American students.

## 1 Introduction

Traditionally, physical education has relied on direct instruction (Dervent et al., 2021). This approach places the teacher at the center of the instructional/learning process by making them responsible for virtually all decisions about lesson content, objectives, management, and student responsibilities. Students are required to repeatedly perform movements as instructed by the teacher (Metzler, 2017). Moreover, this model's overemphasis on developing students' techniques has resulted in students reporting low levels of motivation and learning in physical education (Metzler, 2017). According to Casey and Kirk (2020), traditional physical education methodologies exhibit additional limitations. First, it lacks inclusivity, inadvertently excluding a significant number of youths from reaping its potential educational benefits. And second, this approach is characterized by brief instructional units, often encompassing merely four to six sessions before transitioning to a new activity. Launder and Piltz (2013) emphasized the critical need for ample opportunities for students to practice skills in an authentic manner. This authentic practice is vital for nurturing motivation in physical education, an aspect often overlooked in traditional physical education. Echoing this sentiment, Ward and Lehwald (2017) noted that without the proper development of the essential game-play skills, students' motivation to engage in sports diminishes, further underscoring the drawbacks of traditional instructional methods used in physical education.

To address this issue, Siedentop (1994) developed the Sport Education model (SE), a student-centered curriculum and instruction approach intended to provide youths with more authentic, complete, and enjoyable sport experiences than typically seen in traditional physical education classes (Siedentop et al., 2020). SE has three central goals: To develop students' competence and literacy in and enthusiasm for sport. It has six central features, including (a) roles other than player, (b) working together on persisting teams, (c) keeping records, (d) formal competition in games modified to be small-sided and developmentally appropriate, (e) organizing units into longer seasons, and (f) culminating event and festivity (Siedentop et al., 2020).

SE has been thoroughly examined in the United States and other Western countries, such as Australia, the United Kingdom, Spain, and Portugal, as well as in several non-Western countries, including Korea, Russia, and China (Hastie et al., 2011; Araújo et al., 2014). There is strong evidence that the model can motivate students in physical education (Wallhead and Ntoumanis, 2004; Spittle and Byrne, 2009; Wallhead et al., 2014; Chu et al., 2022; Méndez-Giménez et al., 2022). Students also have been reported to have a highly positive perception of SE, and they prefer it over traditional physical education (Siedentop et al., 2020).

### 1.1 Impact of the SE on students' motivation

Perlman (2011) demonstrated that SE positively influenced students' psychological needs. Researchers have also specifically explored the impact of SE on students' motivation. In a study conducted in the US, Wallhead et al. (2014) examined the effects of SE on high school students' motivation for leisure physical activity and for physical education in one school. In a comparison school, one teacher taught using traditional teacher-directed approach. The students in the SE group reported significantly greater increases in enjoyment and perceived effort of SE compared to the students in the comparison school.

These findings were similar to those reported by Wallhead and Ntoumanis (2004). Students in SE class, also in a US context, reported significant pre- to post-intervention increases in enjoyment, perceived effort, and motivation during physical education. In contrast, students in traditionally taught classes did not report any significant changes in motivation.

Regardless of the country where the studies were completed, the researchers' findings were consistent. Spittle and Byrne (2009) investigated the influence of SE on student motivation (intrinsic/extrinsic motivation, goal orientations, and perceived motivational climate) in secondary physical education in Australia. Compared to students in the traditional teacher-directed approach class, students in SE class had significantly higher levels of perceived competence, mastery climate, and task orientations, with the traditional class condition declining significantly from pre- to post-test in motivation compared to SE condition. There were no significant differences concerning enjoyment/interest, ego orientation, effort/importance, or performance climate.

### 1.2 Student perception of SE

Perlman and Goc Karp (2010) investigated students' and teachers' perceptions and experiences within two consecutive seasons of SE framed through the perspective of Self-Determination theory (Ryan and Deci, 2000). This theory suggests that providing students with a social context that promotes the innate and critical psychological needs of competence, relatedness, and autonomy can affect the control they feel over situations and their motivation. A diverse class of 24 high school physical education students from the U.S. participated in two consecutive seasons of invasion games using SE. A case study approach was used to collect the data through teacher and student interviews, as well as researcher field notes. Results showed the primary emergent themes of teacher and student experiences were winning as a team, social support, and the positive impact of SE on self-determination. Social support present in SE is further explained through two sub-categories: sportspersonship/fair play and inclusion. In addition, it was shown that certain aspects of SE (e.g., an effective gameplay rubric and team affiliation) support students' self-determination and psycho-social needs.

Hastie and Sinelnikov (2006) examined the perceptions and participation of a group of Russian secondary school students who played in a basketball season taught with SE. The season consisted of 18-lesson seasons and was completed by 37 students from two classes. Students found SE season interesting, they enjoyed new roles this model offered (e.g., coaching) and they enjoyed developing team affiliations.

This aligns with the findings from similar studies in Spain by Gutiérrez et al. (2013) and Portugal (Mesquita et al., 2016).

Méndez-Giménez et al. (2022) surmised that this was a consequence of how the SE's longer season provides the students more opportunity to learn, to work cooperatively, and experience sport in a more authentic way.

Thus, SE has been studied extensively since its inception in the early 1980's. There are now over 100 published studies demonstrating its positive outcomes among teachers and students across learning domains (Siedentop et al., 2020). However, no studies have been identified that assess SE's effectiveness in any Arab country. SE is a student-centered learning approach, and some studies question the effectiveness and usability of student-centered approaches in non-Western countries because of cultural barriers and different views of the role of teachers (Thanh, 2010). Moreover, most international studies have attempted to determine the effectiveness of this model in their respective countries. However, no study was found that had attempted to compare its effectiveness in various respective countries to its effectiveness in the U.S. context where this model was developed.

Since 2004, the Ministry of Education (MOE) in Kuwait has actively engaged with a wide range of international consulting organizations and firms, aiming to overhaul its educational systems and curriculum. This effort included partnerships with the World Bank, UNESCO, the British Council, the Centre for British Teachers, McKinsey and Company, Tony Blair Associates, and the National Institution (Alhashem and Alhouti, 2021). More recently, a notable collaboration with the World Bank again in 2015/2016 led to the recommendation of the Competence Based Curriculum, a student-centered curriculum implemented in schools across Kuwait. However, just 3 years after its implementation, the curriculum was discontinued and criticized for its poor fit within the Kuwaiti context (Sadeq et al., 2021). The primary reasons for its perceived inappropriateness were manifold: Resistance from the deeply rooted conservative community, skepticism from parents about the model's efficacy, a lack of local expertise since the model was predominantly developed by international researchers, and concerns regarding the fairness of the curriculum's assessment methods, which significantly departed from traditional approaches.

In addition to these efforts, the MOE's initiatives have faced criticism for not aligning well with the Kuwaiti context (Alhashem and Alhouti, 2021). This has led educational scholars to question the applicability of Western educational strategies in Kuwait. As a result, a comparative study examining the impact of SE on student motivation in both Kuwait and the United States could provide more robust evidence and a more comprehensive analysis than research confined to a single country. Exploring how student motivation varies or aligns between these countries could offer significant insights into the role of cultural and contextual factors in shaping the success of educational models.

This issue is particularly critical in Kuwait and many Arab countries, where teachers are not supposed to independently choose their own preferred curriculum or education model. Instead, all schools and teachers must adhere to the directives from the MOE. Strong evidence demonstrating the effectiveness of SE in Kuwait is essential. If it can be shown that SE has a positive impact on Kuwaiti students' motivation, comparable to its effectiveness with American students, it may prompt the MOE to reconsider its current physical education curriculum or to further investigate the potential of SE in Kuwait. This may also encourage more local and regional research into the benefits of SE. Additionally, the study could benefit international researchers by providing a comparative analysis of the effectiveness of SE between Kuwaiti and American students.

Therefore, the purpose of this study was to examine the impact of SE on Kuwaiti secondary school students' motivation in PE and to compare the impact of SE on student motivation between Kuwaiti and American secondary school students. The study was aimed at the following three questions.

(Q1) What is the impact of SE on Kuwaiti Secondary school students' motivation in physical education?
(Q2) Is there a significant difference in the motivation of students from Kuwait and U.S. following a SE season experience?
(Q3) What are Kuwaiti and American students' perceptions of SE?

## 2 Materials and methods

### 2.1 Settings and participants

In accordance with standard human subjects protection protocols in the U.S. and Kuwait, human subject approval was obtained from the University in the U.S. and the school principals in both the U.S. and Kuwaiti schools. Parent consent and student assent forms were sent out and collected before the initiation of the project.

#### 2.1.1 School in Kuwait

In the study conducted in Kuwait, there were 37 sixth-grade student participants (aged 10–11 years) from a single private school. Two classes were selected according to teacher and class availability. These classes were randomly assigned to different teaching conditions. The first class, consisting of 18 students (12 boys, six girls), was taught using SE. The second class, comprising 19 students (13 boys, six girls), was taught using a traditional teacher-directed instructional approach.

#### 2.1.2 School in the Southwestern U.S.

In the Southwest US, the study involved 33 students from sixth and seventh grades (aged 11–12). Similar to the approach in Kuwait, two classes were selected, also based on teacher and class availability. One of these classes, taught using the SE approach, included 16 students (12 boys, five girls). The other class, which was taught using traditional teacher-directed instructional approach, had 15 students (12 boys, four girls).

### 2.2 Study design and procedures

A replicated mixed methods quasi-experimental pre-test and post-test design. The embedded mixed design was used. In this study, a quantitative approach to data collection was used, while a qualitative approach was embedded to provide additional insight to the quantitative findings (Ivankova and Creswell, 2009; Creswell and Creswell, 2017). The study took place in Kuwait, and was then replicated in Southwestern U.S. in the winter of 2022-23. A pre-/post-test quasi-experimental design was used involving a group of students who were taught via SE and a comparison group of students who were taught using a traditional teacher-directed approach. Both groups were taught 10 basketball sessions, and the study in both countries lasted for 3–4 weeks. Kuwaiti and U.S. lessons lasted 45 and 50 min., respectively. In both countries, four physical education lessons were delivered per week. Group differences were explored between the participant groups for each country and between countries.

In Kuwait, two classes (an intervention class taught using SE and a class instructed using the traditional method) were administered a pre-test using the selected motivation questionnaire. The pre-test was carried out in the 1st week of the research project. A post-test was administered during the last day of the study to determine student motivation changes in both classes after the intervention. All student participants in SE intervention group were interviewed in the last week to explore their perceptions of the model. To ensure consistency in the delivery of SE between its delivery in Kuwait and the US, the study was then replicated using the same procedures in U.S. and taught by the same instructor.

#### 2.2.1 Physical education teacher

The first author served as the instructor for all classes in both Kuwait and the U.S. He had intensive training experience implementing SE. He holds a bachelor's degree in physical education and taught physical education for 6 years in elementary and secondary schools. He also holds certified basketball certificates from the United Kingdom. Teacher content knowledge of the sport may affect student experience in physical education classes (Iserbyt et al., 2016). Therefore, the first author taught both classes (intervention and control) in Kuwait and the US to avoid the teacher effect. Basketball was chosen as the unit of instruction because it is very popular in both countries and is taught in schools in Kuwait and U.S.

#### 2.2.2 Sport Education conditions

The students were taught basketball using SE and its six features. The lessons were created based on the textbook *Complete Guide to Sport Education* (Siedentop et al., 2020). There was a mini-season during which the students were assigned to teams for which they played until the end of the intervention. Beyond the role of player, they were instructed to adopt selected non-playing roles (e.g., coach, fitness trainer, referee, etc.). The specific season consisted of three phases: One teacher-directed practice, one preseason scrimmage and student-led practice, and a formal competition. In the 1st week of the intervention, the instructor formed a team selection committee from among the students in the SE group. The season block used for this SE season is presented in Table 1.

**Table 1:** SE season plan.

**Lesson**	**Sport Education**
1	Introduction to the model, what it is, what roles the students will play during the league season and the importance of fair play were discussed. Basketball activities were led by the teacher.
2	How points are earned and how the winning team is determined, as well as duties and team responsibilities were discussed. Non-playing roles were practiced.
3	The students were assigned to teams of balanced ability. Teams' names and students' roles were chosen by students. Students then practiced together under teacher supervision.
4	Students led the activities. All the teams' members, under the coach's direction in every team. Students started with a warm-up and practice with their teams, followed by scrimmages with opposing teams. Team responsibilities were determined (e.g., equipment manager, statistician, referee, and photo taker). The students practiced, but no points were awarded yet.
5	Students directed the instruction, same procedure just like day 4 was followed.
6	Beginning of the league. The coaches led the instruction. The teams then played matches and started earning points by winning matches and from other duties. The players' team responsibilities (e.g., equipment manager, statistician, referee, and photo taker) were checked, and points were awarded for meeting responsibilities.
7	Points were awarded to the teams. The coaches led the instruction. The teams played matches and earned points. The players' team responsibilities were checked, and points were awarded for meeting responsibilities.
8	Students continued the season, and followed day 7 procedures.
9	Students continued the season, and followed day 7 procedures.
10	Culminating event and festivity.

Students with prior experience in SE tend to be more adaptable and confident in fulfilling the diverse roles and responsibilities associated with SE, as highlighted in research by Sinelnikov and Hastie (2010). In line with this, the first author confirmed with the two classes' physical education teachers and students from both countries whether they had been previously taught using SE. They reported having no prior experience with SE.

#### 2.2.3 Traditional physical education class conditions

Classes in this group were characterized by teacher-directed drills and focused on repetitive drills of technical skills (e.g., passing, dribbling, and shooting) commonly seen in the traditional instruction approach. No changes were made to the classes comprising warm-up activities or skills development work. Upon completing the drills, the students engaged in 10 min of full-sided games. In these games, the students were put on different teams. The teacher was in charge of choosing the content, managing tasks, presenting and structuring them, guiding interactions, setting the pace, and conducting evaluations (Metzler, 2017). The teacher also determined group and team formations, which varied for each session (see Table 2).

**Table 2:** Traditional instruction.

**Lesson**	**Traditional instruction**
1	Dribbling
	Teacher-led warm-up and practice
	Individual and partner dribbling drills
	5 min of a full-sided game
2	Shooting
	Teacher-led warm-up and practice
	Individual and partner free and lay-up free-throw shooting drills
	10 min of a full-sided game
3	Shooting
	Teacher-led warm-up and practice
	Individual and partner jump and lay-up shooting drills
	5 min of a full-sided game
4	Passing
	Teacher-led warm-up and practice
	Individual and partner drills for chest and overhead passes
	10 min of a full-sided game
5	Passing
	Teacher-led warm-up and practice
	Push pass and off-the-dribble passing drills, with a partner/partners
6	Defense
	Teacher-led warm-up and practice
	Defensive drills with partners
	10 min of full-sided games
7	Offense
	Teacher-led warm-up and practice
	Offensive drills, done individually and with partners
	5 min of two full-court games
8	Rebounding
	Teacher-led warm-up and practice
	Rebounding drills with partners
	5 min of full-sided games
9	Competition
	Teacher-led warm-up followed by a full sided competition
10	Competition, winning team announced
	Teacher-led warm-up followed by a competition, announcement of the winning team (no festivity)

#### 2.2.4 Validity and fidelity of the intervention

The lesson plans that were written for the SE intervention groups were validated by an expert teacher with extensive experience in teaching SE and having introduced pre-service teachers to the SE. Changes and recommended revisions by the expert were made.

Hastie and Casey (2014) have emphasized the importance of providing details on the fidelity of educational interventions. The fidelity of the intervention was validated using an SE benchmark observational instrument (Sinelnikov, 2009). Two pedagogy experts in Kuwait and one in the US observed three of the intervention classes. The interrater reliability on the observation instrument indicated that the intervention was taught 100% of the observed time.

### 2.3 Data collection

Three data collection tools were used. They included the Intrinsic Motivation Inventory (IMI), small group interviews, and field notes.

#### 2.3.1 Intrinsic Motivation Inventory (IMI)

Intrinsic motivation for physical education was measured using the Intrinsic Motivation Inventory (IMI; Ryan, 1982). This instrument has been shown to produce reliable and valid scores in a similar sample of students (McAuley et al., 1989). The IMI consists of 18 questions to measure enjoyment/interest, perceived competence, effort/importance, and pressure and tension. The students were instructed to consider the statement “Playing the basketball game was fun...” They were asked to read the 18 questions on the questionnaire and indicate the extent to which they agreed with each statement using a scale of 1 (strongly disagree) to 7 (strongly agree).

Sousa and Rojjanasrirat (2011) provided guidelines for the cross-cultural validation of instruments. The first author adhered to these guidelines to translate, adapt, and validate the English version of the IMI questionnaires, thereby establishing culturally equivalent versions for Kuwaiti students. First, the instruments were translated by two Arabic-speaking university professors, one with a Ph.D. in Teaching English to Speakers of Other Languages (TESOL) and one with a U.S. Ph.D. in PE. Second, a certified English translator compared the two translated versions with the original questionnaires. Ambiguities and discrepancies were resolved among the three translators and the first author, and full agreement was reached among all.

Third, the two translated (i.e., English) copies were blindly translated back into Arabic language by two different Arabic-speaking university professors, both with a Ph.D. in English literature. Fourth, the first two Arabic-speaking university professors, the certified English translator, and the first author compared the back-translated questionnaires with the originals and found no ambiguities or discrepancies.

Finally, to ensure the content validity of the instrument, a panel of experts reviewed the translated questionnaire and unanimously agreed on its content validity without suggesting any changes. Additionally, a pilot test was conducted in two classes not included in the study to assess face validity. During this phase, students were asked to rate the clarity of the questionnaire as 0 (unclear) or 1 (clear). Remarkably, the inter-rater agreement exceeded 92%, surpassing the recommended 80% threshold set by Sousa and Rojjanasrirat (2011).

#### 2.3.2 Interviews

Five small group interviews using a focus group format were conducted with all student participants in the SE group in both countries (Fontana and Frey, 1994). Each interview consisted of 3–5 students and lasted 25–30 min. This interview technique ensures interaction among students and has several other advantages: (a) the provision of social support from peers; (b) positive interviewee attitudes toward interviews due to the presence of friends; and (c) the emergence of a conversational format rather than a formal interview, as the interviewer acts as a facilitator or mediator to guide the discussion and encourage students to express and debate their views (Tammivaara and Enright, 1986).

One potential limitation of group interviewing is that some students may feel hesitant to share feelings or information in front of their classmates. To overcome this, we followed Carlson and Hastie (1997) suggestion, asking students to choose friends they felt comfortable with for the group interview. Students were then reminded that these were group discussions and that conversations should not be dominated by one student.

Semi-structured interviews using a general interview guide were carried out to explore the perceptions of American and Kuwaiti secondary school students regarding SE, to determine if they perceive SE differently. The interview guide was developed through reading literature on students' perceptions of SE (e.g., Hastie and Sinelnikov, 2006; Gutiérrez et al., 2013). Students in the group interviews were asked about their views on participating in the Sport Education season, how they differentiated SE from normal physical education, whether they liked being on the same team throughout the season, and their perception of the culminating festive event. All interviews were recorded using a voice recorder (Sony ICD-UX570). The first author manually transcribed all the interviews. Manual transcription of interviews is beneficial as it facilitates a thorough immersion in the data (Byrne, 2022). All interviews were transcribed orthographically to capture nuances such as pauses, breaks, and tonal variations from both the interview participants and the interviewer (Byrne, 2022). Given that the research team included researchers from both the US and Kuwait, all the transcribed interviews for the Kuwaiti participants were translated into English by the first author. A professional bilingual translator from Kuwait then compared the translated interviews with the original versions (McKenna, 2022). Any suggested changes by the two translators were implemented after discussion with the first author, who is from Kuwait.

For all data related to individual students, pseudonyms were used in place of their real identities to ensure anonymity.

#### 2.3.3 Field notes

During the 10-class basketball unit, the researcher made thorough observations of the engagement of students, with a focus on perception and enjoyment. A total of 33 logs of field notes were made to complement interview data.

### 2.4 Date analyses

The analysis started first with independent *t*-tests to determine whether there were differences in the pre-test scores across the four IMI dimensions between the Kuwaiti SE and traditional PE groups, as well as between the Kuwaiti SE and American SE groups. No significant differences in the pre-test scores between groups within each country and between SE groups across the two countries were found. Based on the Shapiro–Wilk test for data normality, data were found to be normally distributed. This led to the use of parametric statistics. Levene's test results did not show any homogeneous variances.

Cronbach's alpha coefficient was calculated to determine the internal reliability of each dimension of the IMI for each country, group, and time. Cohen's d (Cohen, 2013), was employed to show the extent of differences in group averages. The scale of the effect was assessed using Cohen's benchmarks: *d* = 0.2 for a small effect, *d* = 0.5 for a medium effect, and *d* = 0.8 for a large effect.

First, to compare the differences in the impact of SE on students' motivation in Kuwait between the two instructional conditions, a series of 2 (Group) X 2 (time) repeated measures analyses of variance (RM-ANOVAs) were performed. In the case of a significant interaction, a paired *t*-test was performed for both the intervention and control groups to assess whether the observed changes within each group were statistically significant. Second, to compare the effects of SE on motivation between Kuwaiti and American students, a series of 2 (countries) × 2 (groups) × 2 (times) mixed ANOVAs were conducted. If a significant interaction effect was found, an independent *t*-test was performed to compare the post-test results between the two countries.

Before analyzing the effects of the intervention between groups and between countries, Bonferroni adjustment has been made to the alpha = 0.006 because of the performing of multiple ANOVAs. Moreover, because of performing multiple *t*-tests, a Bonferroni adjustment was made to the alpha = 0.002.

#### 2.4.1 Qualitative data analysis

A thematic analysis technique was employed in this study, found to be effective for gaining insights into participants' perceptions, thoughts, and experiences by identifying and examining recurring patterns or themes within the data (Kiger and Varpio, 2020). Responses were initially coded and then systematically categorized into themes (Smith et al., 1995).

The data analysis began with a thorough familiarization phase. The first author read through the entire dataset several times, aiming to identify relevant information pertaining to the research question (Byrne, 2022), and to recognize emerging patterns (Male, 2016). This comprehensive review laid the groundwork for the subsequent coding phase.

Subsequently, a systematic data coding procedure was implemented, employing an inductive coding technique. This approach allows knowledge to naturally emerge from participants' perspectives and experiences, ensuring that themes originate organically from the data (Frith and Gleeson, 2004). In this process, two members of the research team carefully coded the data without any pre-set categories, allowing the data to guide the emergence of codes. For example, codes such as “enjoyment in SE” and “pressured in traditional physical education” were identified directly from participants' responses, and were written in the transcripts' margins.

After coding all the data, the codes were then carefully analyzed to form overarching themes. This stage involved a thorough examination of how each code represented an aspect of the students' perceptions. Two members of the research team met to review and deliberate on their initial coding efforts and subsequently grouped codes that shared a common thread, thereby forming distinct themes. For instance, the codes “enjoyment in SE” and “pressured in traditional physical education” contributed to the formation of a broader theme like “Perspectives on Past vs. New Experiences.”

Once all data had been coded, an analysis of the codes was conducted to identify patterns that could be grouped into themes (Byrne, 2022). The research team collectively reviewed these themes alongside the coded data and the dataset, deliberating on which themes to include in addressing the research question.

The final phase involved the refinement and naming the themes. To achieve this, the dual criteria suggested by Patton (1980) were applied. The research team reviewed all the themes to ensure that each theme offered an internally consistent and coherent interpretation of the data, distinct from the interpretations from other themes, and effectively addressed the research question. Any themes that did not align with the data were excluded following discussions between the research team members. Additionally, the research team collaboratively determined the most suitable labels for the identified themes. Adjustments were made to the initial theme labels to more accurately represent the students' experiences. For example, initially, the theme was named “Social Interactions in SE,” reflecting the general nature of students' social experiences in SE. However, a more thorough analysis by the team revealed a strong emphasis on the positive impact and value of these interactions. Consequently, the theme was refined to “Appreciation of the Social Aspect of SE.” This name was selected to more accurately reflect the depth of students' responses, highlighting not only their positive experiences but also their explicit appreciation and perceived value of the social elements in their SE experiences.

#### 2.4.2 Qualitative data trustworthiness

The trustworthiness of the qualitative data was assessed in multiple ways. Member checks of themes were conducted with the participants. Data triangulation was used by comparing intervention findings across student groups as well as with the field notes. Peer review was used by having two researchers review the transcriptions and identify themes independently. They then negotiated and agreed on final themes. Finally, a search for negative cases searches was conducted.

## 3 Results

Internal consistency reliability scores (Cronbach's Alpha) ranged between 0.72–0.88 for all subscales pre and post. Which met the threshold according to Nunnally's (1975) cutoff of 0.70. Cronbach's alpha results are shown in Table 3.

**Table 3:** Cronbach's alpha coefficients for the four IMI subscales at pre-and post-test.

**Questionnaire**	**Subscale**	**Pre-test α**	**Post-test α**
IMI-Kuwait	Interest/enjoyment	0.76	0.73
	Perceived competence	0.79	0.82
	Effort/importance	0.85	0.88
	Pressure/tension	0.75	0.76
IMI-US	Interest/enjoyment	0.76	0.84
	Perceived competence	0.73	0.88
	Effort/importance	0.82	0.72
	Pressure/tension	0.82	0.76

α, Cronbach's alpha.

Table 4 includes descriptive statistics for both countries and both groups for all the measures of motivation at pre-and post-test. They address the study's specific research questions.

**Table 4:** Descriptive statistics for the measures of motivation.

**Country**	**Subscale**	**Group**	**Pre-test**		**Post-test**	
			**M**	**SD**	**M**	**SD**
KW	Interest/enjoyment	SE	2.83	0.453	4.68	0.509
		Traditional	2.65	0.421	2.71	0.389
	Perceived competence	SE	2.76	0.481	4.17	0.802
		Traditional	2.66	0.411	2.70	0.418
	Effort/importance	SE	2.57	0.363	4.72	0.398
		Traditional	2.70	0.523	1.86	0.422
	Pressure/tension	SE	2.84	0.458	4.14	0.451
		Traditional	2.93	0.731	2.16	0.647
U.S.	Interest/enjoyment	SE	2.73	0.540	4.81	0.441
		Traditional	2.64	0.401	2.73	0.343
	Perceived competence	SE	2.93	0.428	4.26	0.729
		Traditional	2.49	0.345	2.57	0.967
	Effort/importance	SE	2.81	0.505	4.84	0.394
		Traditional	2.63	0.581	4.20	0.414
	Pressure/tension	SE	3.08	0.594	4.36	0.607
		Traditional	2.91	0.652	2.46	0.632

IMI-KW represents the IMI scores for the school in Kuwait; IMI-U.S. represents the IMI scores for the school in U.S.

Addressing the first research question:

**Q1: What is the impact of SE on Kuwaiti students' motivation in physical education?**

Based on RM-ANOVA, there was a significant Group X Time interaction [*F*_(1,35)_ = 112.5, *p* < 0.001, η = 0.763] for the interest-enjoyment dimension. Based on the follow-up paired *t*-tests, there was a significant change in motivation in SE group in Kuwait [*t*_(18)_ = −11.66; *p* = < 0.001; *d* = 0.69]. With no significant change was found in the control group in Kuwait [*t*_(17)_ = −2.55; *p* = 0.010; *d* = 0.09].

A significant Group X Time interaction for the perceived competence dimension was found [*F*_(1,35)_ = 31.1, *p* < 0.001, η = 0.471]. Furthermore, there was a significant change in perceived competence among SE group in Kuwait [*t*_(18)_ =−6.12; *p* < 0.001; *d* = 0.96]. However, no significant change was found in the control group in Kuwait [*t*_(17)_ = −1.84; *p* = 0.041; *d* = 0.07].

For the effort and importance dimension, a significant Group X Time was found [*F*_(1,35)_ = 667.7, *p* < 0.001, η = 0.950]. Based on the Follow-up paired *t*-tests, there was a significant change in effort and importance among SE group in Kuwait [*t*_(18)_ = −38.9; *p* < 0.001; *d* = 0.24] and a significant decrease in the control group in Kuwait [*t*_(17)_ = −8.35; *p* = < 0.001; *d* = 0.42].

A significant Group X Time effect for the tension and pressure dimension was found [*F*_(1,35)_ = 447.9, *p* < 0.001, η = 0.9282]. Based on the Follow-up paired *t*-tests there was a significant change in this dimension for Kuwait's SE group [*t*_(18)_ = −42.4; *p* < 0.001; *d* = 0.13] and a significant decrease in Kuwait's control group [*t*_(17)_ = 7.08; *p* < 0.001; *d* = 0.34].

Analysis of effect sizes in the SE condition reveals a medium effect size for interest-enjoyment, a large effect size for perceived competence, and small effect sizes for the effort and importance dimensions as well as for tension and pressure.

**Question 2: What is the Difference in the Impact of SE on Kuwaiti vs. American Students' Motivation?**

Based on Mixed-ANOVA results, no Country X Group X Time interaction was found for the interest-enjoyment dimension [*F*_(1,66)_ = 0.557, *p* = 0.458, η = 0.008], perceived competence dimension [*F*_(1,66)_ = 0.094, *p* = 0.760, η = 0.001], and pressure and tension dimension [*F*_(1,66)_ = 0.249, *p* = 0.035, η = 0.208]. This shows that interest-enjoyment, perceived competence, pressure and tension were not significantly different between the Kuwaiti and American SE groups. However, there was a significant Country X Group X Times interaction for the effort and importance dimension [*F*_(1,66)_ = 0.142.7, *p* < 0.001, η = 0.684]. Based on the follow-up independent *t*-test, there was no significant difference found between the Kuwaiti and American SE groups in the post-test [*t*_(35)_ = −0.94; *p* = 0.350; *d* = 0.03]. However, there was a significant difference observed between the control groups in the post-test scores with higher scores for the American students [*t*_(31)_ = −15.98; *p* < 0.001; *d* = 0.41].

**(Q3) What are Kuwaiti and American students' perceptions of SE?**

Five themes were drawn from the interviewees' responses:

- Past vs. new experience.
- Appreciation for the culminating event.
- Appreciation for the socialization aspect of SE.
- Appreciation for peer tutoring.
- Appreciation for the matched ability teams, and the ability to contribute to one's team.

### 3.1 Perspectives on past vs. new experiences

Consistent with the findings of previous studies, both Kuwaiti and American students enjoyed SE and preferred it to traditional PE. When the first author asked the students about their perceptions of SE and how it differed from typical physical education, the majority of students in both Kuwait and the US commented that they have had bad experiences in physical education and that they preferred SE to normal physical education.

In relation to their previous experiences in physical education, the majority of both Kuwaiti and American students perceived physical education to be pressured and stressful, with a lack of enjoyment due to the overemphasis on winning. For example, Fatima, a Kuwaiti girl in the SE group commented: “PE was sort of pressured, I just got yelled at. If we lost a point, we kept getting yelled at, so I always preferred to do activities in PE rather than playing a sport or a game.”

Katie, an American girl in the same group stressed the same point: “physical education was sometimes intense, and being a girl in a team of skilled students makes it hard for us to enjoy it. We don't do things well, and this makes us feel unwanted. We don't get chosen because every team wants to win, so why should they pick us?”

In relation to the new experience with SE, both Kuwaiti and American students perceived SE as fun and interesting. Being with the same team throughout the season and having a long season of play made it interesting as it helped them improve. Ahmad, a Kuwaiti boy commented:

“SE was fun, with everybody like working together and having a great time, and everyone is having fun here. It's not like everyone's miserable; it's like everyone's having fun and enjoying themselves. We practice together as a team, and we all have the same goal. We care about each other, and that's good.”

Yousif, another Kuwaiti boy commented, “I love that we stay in the same team, and you put us in matched teams, so we all have a chance to win.” Yaqoub, another Kuwaiti boy stressed the same point:

SE was interesting and different from normal PE. We didn't move from one sport to another; we played basketball for a long time, and that helped us to improve. I scored some points because I improved a lot, and that was fun. We had enough time to practice, and that helped us to improve and really enjoy playing.

Chris, an American boy made a similar comment: “I definitely like SE because, instead of switching back-and-forth, you stay with one team throughout the season. It was fun, and helpful.” Cameron, another American boy stressed the same point, saying:

Being on the same team has helped us, as we all now understand each other, and we know what might work for us. We know each other because we have been on the same team since the beginning of the season.

Consistent with the findings of previous studies, the Kuwaiti students enjoyed non-playing roles, and they stated that these helped them to improve their skills. Being the referee was the favorite non-playing role of most of the Kuwaiti and American students interviewed. Hamad, a Kuwaiti student commented, “I liked being the referee; it helped me to see the game better and to identify the weak areas in teams.” Khalid, another Kuwaiti said, “Refereeing helped me know how to move in the game and what to do.”

Consistent with other studies, both Kuwaiti and American students enjoyed non-playing roles, especially coaching and refereeing, stating that these helped them to improve their skills and understand the rules. For example, Ahmad, a Kuwaiti boy stated, “Being a ref gave me an opportunity to observe and to know what to do in the game.” Similarly, Jarrah, another Kuwaiti boy said, “Being a ref helped me to know more about the rules so I don't make mistakes, and this helps me to improve as if I don't make mistakes, my team improves.”

Similarly, Audrey, an American girl stated, “Being a ref helped me to see the other team's strengths and weaknesses, so we could practice how to beat the other team.” Hannah, an American girl stated, “I liked being the coach, because I could prepare what I would coach my team when we practiced.”

### 3.2 Appreciation for the culminating event

Both Kuwaiti and American students perceived SE to be very close to the real game, and they thought the “festivity” of the culminating event was fun and interesting. Yousif, a Kuwaiti boy commented, “The last event was fun. It was like the Kuwati basketball league.” Khalid, another Kuwaiti boy commented similarly: “The last event was fun, and I liked that we started the game by playing the national anthem; it made it so real.”

The Kuwaiti students also liked the award ceremony and appreciated that awards were given to more than just the best player, Shahad, a Kuwaiti girl commented “Being awarded trophies and medals was exciting and fun.” Ahad, another Kuwaiti girl said, “I liked having awards such as the most improved player or the fairest player. It showed that someone appreciated the effort we put in.”

The American students also perceived the last event as fun and authentic, Jason an American boy said “It was fun and similar to NBA.” Michael, another American boy commented, “The last event was such fun, it was so emotional.”

Hannah, an American girl made the interesting point that the awards ceremony was not only interesting, but it also gave her an equal opportunity to be awarded. “Normally in PE, the best student is awarded, but as girls, we can never be as good as the boys, so it is fair to make awards for the best girl player and the most improved player. That way, everyone can win an award.”

Based on the field notes, students enjoyed the culminating event and that they were still talking about it in school days after the season was over. The students also talked to other teachers in school about the awards they had received.

### 3.3 Appreciation of the Social Aspect of SE

The interviews showed that the Kuwaitis particularly emphasized the social dimensions provided by SE. All the Kuwaiti students interviewed stated that they had stayed on the same team throughout the season and that practicing and competing together had helped them socially. Meshal, a Kuwaiti boy commented, “We were in a team together for weeks, we trained, we discussed plans, we competed together, everyone was nice and encouraging, everyone wanted to cheer me up, and now I am friends with some of my team members.” Khalid, another Kuwaiti student agreed, making the following comment:

“Being together throughout the season helped us to talk to someone new. I used to play with XX and XX, as we loved to play together, and we always used to be on the same team. In this class, I was put into a new team. We practice and play together, we encourage each other, and we play together during recess. I liked that we got to know new students and be friends with them. We are more than a team now; we are friends.”

Based on field notes, when starting to teach SE, in the initial classes before teams were formed, the students tended to talk and play with a specific group of students only. If someone's friend was missing due to absence, they tended to lose their enthusiasm for playing, and in some cases, they refused to play at all. However, as the season proceeded, this behavior disappeared, despite the fact that everyone was in a team with students who had not been their friends before.

### 3.4 Appreciation for peer tutoring

In SE, Students are given the opportunity to take on various leadership roles (e.g., team coach, team fitness conditioning coach, and team captain; Siedentop et al., 2020). These represent peer-to-peer instruction (also referred to as peer tutoring). In relation to peer tutoring, both the Kuwaiti and American students liked being coached by their peers rather than teachers. Jarrah, a Kuwaiti boy commented: “I feel, yes, being taught by our friends was more fun and useful than being taught by the teacher; we know each other better.” Nasser, another Kuwaiti boy stressed the same point: “We understand each other's feelings better, better than the teacher does.”

Similarly, George, an American student, expressed a positive perception of being taught by their peers instead of a teacher. One American student stated, “Teachers normally teach all the students the same, but our strengths and weaknesses are different, so being coached by our peers helps us to improve more.” Logan, another American boy said, “Being taught by our friends helped us to improve, and it gave us more time to practice the skills we needed.” Cameron, an American boy, who had been a coach, made an interesting point. He stated that peer tutoring is important, as in every team, no one wants a weak student, so peer tutoring makes it possible to spend more time improving the weak students:

“You're only strong as your weakest link. If your weakest link is improving, the whole team improves. All the girls in my team improved because I spent more time with them, trying to improve their skills, and a teacher would not have spent that much time on a few students.”

### 3.5 Appreciation for the mixed ability teams, and the ability to contribute to one's team

All the Kuwaiti students interviewed enjoyed participating in matched ability teams, and they liked the contribution they had made to their team, despite their own basketball skills level. As Shahad, a Kuwaiti girl, noted, “It was fun. I liked that all teams were almost equal, so everyone was important. It was not like before, when all the good students were in one team.” Fatima, another Kuwaiti girl commented, “Everyone was trying his best. It is fair when every basketball team has two good and two average students, and we all can help our team.

Some of the Kuwaiti students interviewed were asked how important they felt the scoring system in SE was in helping them to contribute to their team even though they might have been less skilled in basketball. The responses given included the following: “Although I am not the best, I helped my team by coaching them,” and similarly, “I felt very important to my team. I helped them to get fair points.”

The American students interviewed made no different points here. They commented that they liked being in the mixed abilities team because it was fair and that this made the season very exciting because the scores of every team were very close, and the majority of them believed that they had contributed to their team's success. Audrey, an American girl, commented, “It was fun. All the teams were equal, and every day, when you showed us our scores, we didn't know who would be the winner.” Chris, an American boy stated:

“The teams were almost equal, and we all did our best because we wanted to win. I was always there, busy keeping the vibe going and keeping spirits up, so no one was feeling sad, and everyone was happy.”

## 4 Discussion

The purpose of this study was to examine the impact of SE on Kuwaiti secondary school students' motivation in PE and to compare the impact of SE on student motivation between Kuwaiti and American secondary school students. The SE group showed significant improvement from pre- to post-test intervention in various aspects of motivation, specifically interest-enjoyment, perceived competence, effort importance, and tension and pressure. Conversely, the traditional PE group showed no improvement in any aspects of motivation. Groups were not statistically different in their motivation prior to the implementation of the intervention, which is of particular importance because the students in the two groups were not subjected to random assignment.

This significant improvement in SE students' motivation is consistent with prior studies in which students were taught using SE (MacPhail and Kinchin, 2004; Wallhead and Ntoumanis, 2004; MacPhail et al., 2008; Spittle and Byrne, 2009; Perlman, 2010; Gutiérrez et al., 2013; Wallhead et al., 2013, 2014). SE enhances enjoyment in students who show negative attitudes toward PE by building their connections with peers and strengthening team affiliations, which is unseen in traditional PE classes (e.g., Perlman, 2010). Moreover, the enjoyment that SE offers could be explained by the opportunities it provides for students to be involved in the educational experience by allowing them to assume leadership roles in their teams (Grant, 1992; Carlson and Hastie, 1997). The increase in enjoyment can also be attributed to the effectiveness of the SE in meeting the needs of various groups of students, such as those who are less skilled, highly skilled, or less popular (Carlson, 1995; Alexander et al., 1998).

Kuwaiti SE students reported a significant increase in perceived competence. One of the overarching goals of SE is to help students become competent in sports (Siedentop et al., 2020). A competent sportsperson refers to someone with the ability to participate in sports as a knowledgeable game player, having sufficient skill and understanding appropriate to the level of play (MacPhail et al., 2008). Consistent with the results of the current study, Carlson (1995) found that students who were exposed to SE were more competent than students who were taught via traditional physical education methods.

The sufficient length of units enabled by the structure of SE seasons allows more of the focus to be on practice and playing games (Hastie, 1998), which then helps students develop a more positive perception of their competencies. Moreover, in SE, students participate in games with matched-ability teams. Ward et al. (2019) found that grouping students into groups with mixed abilities helped to increase physical activity levels and improve game performance. This might explain the improvement in the students' perceived competence.

Only SE group improved significantly in terms of decreased tension and pressure, which is consistent with Wallhead and Ntoumanis's (2004) study. In SE, students are not constantly told by teachers what to do, and this might help students feel less pressured. Moreover, in the SE, fair play is a central concept, and students have to show social skills, such as respecting other team members and other teams, being supportive and encouraging of peers, and playing by the rules (Vidoni and Ward, 2006). The emphasis on fairness in the SE reinforces the idea that winning is not everything, while in traditional PE, students feel pressure, as the interviewed students stated that in PE classes, they were yelled at. Field notes also showed that the higher-skilled students changed their behaviors throughout the season, and they were the most supportive of their teams.

In addition, SE significantly improved in the effort and importance dimension after the intervention. This is consistent with other studies in which students who were taught via the SE were found to have improved significantly in terms of effort importance (e.g., Wallhead and Ntoumanis, 2004).

In SE, students are organized into longstanding teams throughout the season. This leads to social connections and positive affiliations with the team, which might influence the effort importance students feel toward the team. Hastie (1998) argued that while in traditional physical education, students participate in large-sided games, and some players rarely touch the ball, some players take the court for the entire game. In the SE, students participate in small-sided games with mixed-ability teams, and teams stay and compete together, which forces players to use all team members to succeed. This leads all teams to understand the importance of teamwork and encourages students to do their best. Furthermore, the interviews showed that students in the SE class felt that they contributed to their teams, even when they perceived their skills in basketball as not the best; they felt they did good things in non-playing roles. No significant improvements in this area were found in the traditional PE groups.

Both Kuwaiti and American SE group students preferred SE more than traditional physical education classes due to its features and the opportunity it provided for skill development. In contrast, their past physical education sessions were seen as monotonous, dominated by a few skilled students, often leading to feelings of exclusion. This preference for SE is in line with findings from Russia, Spain, Portugal, and the U.S. (e.g., Hastie and Sinelnikov, 2006; Perlman and Goc Karp, 2010; Gutiérrez et al., 2013; Mesquita et al., 2016).

One thing all the Kuwaiti students stressed was the opportunities that SE provided to interact with other students with whom they were not familiar, as they stated that they worked with and talked to new people and built friendships. According to Evangelio et al. (2018), students' roles under the SE, such as coach, fitness trainer along with team affiliation create the condition that encourages students to interact positively and even develop friendships. Moreover, Rocamora et al. (2019) found that students who were introduced to the SE significantly improved their friendship avoidance goals (i.e., fear of being rejected from teammates).

Kuwait's Ministry of Education previously had unsuccessful experiences with student-led curricula, and Arabs, in particular, may favor teacher-led instruction (Ashour, 2019). Therein lies the significance of the current study in that despite SE being a student-centered approach, there was no significant difference in the impact of SE on the improvement of motivation between US and Kuwaiti students. This might be because SE can better meet the psychological needs of self-determination theory (competence, autonomy, and relatedness) which leads to higher levels of motivation to learn. SE gives students opportunities for autonomy by leaving the leading of activities to them (Wallhead et al., 2010). Moreover, the level of interaction that is necessary in SE provides students with opportunities to enhance relatedness (e.g., Carlson and Hastie, 1997). In SE, students participate in seasons that are longer than traditional PE, with a focus on one sport. This can support students' competency. The traditional approach to PE, in contrast, involves strict regulation of students' actions, which has the potential to hinder their sense of competence, autonomy, and relatedness (Bartholomew et al., 2009).

Although this study contributes to the existing evidence on SE by filling some gaps in the literature on the effectiveness of this model in Arab countries, there are some limitations. First, we employed a convenience sampling method, which means that the results may have been constrained by the absence of a randomized sample. Second, the small sample size limits the generalizability of these findings. Third, the study was conducted in a private, mixed-gendered school in Kuwait; the results of the study cannot be applied to single-gendered schools in Kuwait. Fourth, the first author of the study was responsible for teaching both groups in both countries, which might have potentially introduced some bias. Fifth, the intervention consisted of only 10 sessions, which is less than the recommended 12–15 SE sessions for secondary school students.

Yet, this study also has several strengths. First, the use of both quantitative and qualitative methods (student interviews and field notes) allowed the triangulation of the results of the study and gave a holistic view of the situation. This mixed method study provided comprehensive insights into the changes in motivation that students experienced after being taught using SE. Second, teacher fidelity in teaching using SE was measured and reported. Third, the lesson plans were validated, and the teacher who taught the classes received intensive training in SE. Fourth, the same teacher taught both the intervention and control groups in both countries, which allowed for an accurate comparison of SE without the teacher effect (Browne et al., 2004). Fifth, the researchers recognized external factors that may affect students' motivation, such as the type of sport chosen, weather conditions, and previous exposure to SE. By ensuring that both groups in both countries experienced similar conditions, the validity of the comparison was enhanced. Finally, the instrument used to measure intrinsic motivation (IMI) is widely used in physical education, and studies have shown that the IMI produces reliable and valid scores in a similar youth sample.

Future research should use larger samples and longer units, as well as examine SE at different grade levels (i.e., elementary, high school, and college) and different sports. Future research should also examine SE in single-sex schools because in Kuwait and in the majority of Arab schools classes are single-sex groups. Finally, future research should focus on Kuwaiti teachers' perceptions and attitudes toward SE, especially in that SE is a student-led curriculum, and the leading of activities is left to students instead of teachers. This is especially important in light of the Kuwaiti Ministry of Education's unsuccessful prior experiences with more student-centered approaches.

## 5 Conclusion

In conclusion, Kuwaiti students' motivation (i.e., interest-enjoyment, perceived-competence, effort-importance, and tension-pressure) in physical education can be improved significantly when using SE. Moreover, the same can be said for U.S. students.

The comparison of the impact of SE between Kuwaiti and American students revealed that there was no significant difference in interest-enjoyment, perceived competence, effort-importance, and tension-pressure.

This study adds to the body of literature on SE in two respects. First, it is the first study to examine the SE in Kuwait and provide evidence supporting the effectiveness of SE in improving Kuwaiti students' motivation, which might encourage more research studies to be conducted on the implementation of SE in Arab countries. Second, to the best of our knowledge, this was the first study to attempt to compare the effectiveness of SE in two different countries, which might encourage international researchers examine the effectiveness of the SE, as the results showed that the SE has equal effectiveness in improving students' motivation between Kuwait, and U.S., where the model was developed.

## Data availability statement

The raw data supporting the conclusions of this article will be made available by the authors, without undue reservation.

## Ethics statement

The studies involving humans were approved by Institutional Review Board (IRB)/Arizona State University IRB ID (STUDY00016997) https://era4.oked.asu.edu/IRB/sd/Rooms/DisplayPages/LayoutInitial?Container=com.webridge.entity.Entity [OID [81D70ADF640511ED4F8427D1AF565000]]. The studies were conducted in accordance with the local legislation and institutional requirements. Written informed consent for participation in this study was provided by the participants' legal guardians/next of kin.

## Author contributions

OA: Conceptualization, Data curation, Formal analysis, Methodology, Validation, Writing – original draft. PK: Conceptualization, Methodology, Supervision, Writing – review & editing. HM: Resources, Supervision, Validation, Writing – review & editing.
